# Voided volume < 150 mL on initial uroflowmetry in men with storage symptoms: Is it an unreliable test result or a sign of severe storage symptoms?

**DOI:** 10.1371/journal.pone.0207208

**Published:** 2019-01-07

**Authors:** Sangjun Yoo, Youkyung Lee, Juhyun Park, Sung Yong Cho, Min Chul Cho, Hyeon Jeong, Hwancheol Son

**Affiliations:** 1 Department of Urology, Seoul National University Boramae Medical Center, Seoul, South Korea; 2 Department of Urology, Seoul National University Hospital, Seoul National University College of Medicine, Seoul, Republic of Korea; University of Alberta, CANADA

## Abstract

**Purpose:**

To assess the utility of voided volume on initial uroflowmetry in predicting symptom severity in men with storage symptoms. In addition, we assessed the probability that men would show a voided volume of ≥ 150 mL on uroflowmetry if the examination is repeated.

**Methods:**

Between 2016 and 2017, 352 men with storage symptoms were enrolled in the study. The patients completed the frequency–volume chart and were divided into four groups based on the voided volume. A multivariate analysis was performed to determine the variables affecting voided volume.

**Results:**

The VV was < 68.8 mL in 88 patients (25.0%), 68.9–150 in 89 (25.3%), 150–250 in 87 (24.7%), and ≥ 250 in 88 (25.0%) patients. Although the International Prostate Symptom Score did not differ among the groups, the storage subscore was significantly higher in patients with decreased voided volume (*p* = 0.010). The total number of voids was greater in patients with decreased voided volume (*p* < 0.001), as was the number of nocturnal voids (*p* = 0.007). The maximum voided volume (*p* < 0.001) and 24-h urine output (*p* = 0.003) decreased as voided volume decreased. The proportions of patients with a mean daytime urine output ≥ 150 mL were 30.1%, 43.0%, 64.7%, and 66.7% in each group, respectively (*p* < 0.001). Older age and decreased maximum voided volume significantly affected the voided volume on initial uroflowmetry.

**Conclusions:**

A decreased voided volume on initial uroflowmetry may be a sign of severe storage symptoms in men. This finding is related to older age and decreased functional capacity. In these patients, it is better to perform a careful clinical assessment to diagnose and treat overactive bladder.

## Introduction

According to current clinical guidelines, uroflowmetry (UFM) is a basic assessment test in men with lower urinary tract symptoms (LUTS). [1, 2] Other important examinations include history taking, physical examination, administrations of validated questionnaires, urinalysis, and serum prostate-specific antigen level testing. However, parameters derived from UFM are considered to be clinically reliable only if voided volume (VV) is > 150 mL. [3] Otherwise, it is recommended that UFM be repeated to ensure reliable results before treatment is initiated. [1]

However, in a considerable proportion of patients with LUTS, the VV on UFM is < 150 mL, even when the degree of LUTS is not severe, [4] This may be because 1) the clinicians failed to sufficiently explain the procedure to the patients; 2) the patients failed to understand the procedure, or 3) the patients failed to void during UFM. Alternatively, another reason for insufficient VV on UFM could be severe storage symptoms. However, it can be difficult to accurately determine the reasons for insufficient VV on UFM in daily clinical practice. Thereby, UFM tends to be repeated to ensure that the derived parameters, such as peak flow rate (Qmax) and post-void residual urine (PVR), are accurate.

In contrast, several previous studies have reported that the parameters derived from the repeat UFM do not significantly differ from those derived from the initial UFM. [5–7] That is, the utility of repeat UFM has not been fully elucidated. Moreover, the VV on UFM could be < 150 mL even when the examination is repeated, especially in patients with storage symptoms. In these patients, decreased VV on initial UFM might be a sign of severe storage symptoms. Therefore, we evaluated the clinical utility of VV on initial UFM in predicting the severity of storage symptoms in men who initially visited the outpatient clinic of our urology department. In addition, we determined the reasons for decreased VV on initial UFM and the probability that the patients would show a VV of ≥ 150 mL on UFM if the examination was repeated.

## Materials and methods

### Patient cohort

Among patients who first visited the outpatient clinic of the urology department at Boramae Medical Center between August 2016 and May 2017 because of LUTS, 1185 patients who had storage symptoms and had completed the frequency–volume chart (FVC) were initially considered eligible for this study. Among 1185 patients, 449 female patients, 82 patients with neurologic disease, 5 patients with prostate cancer, 24patients who underwent BPH surgery, 238 patients who did not perform 72-hour voiding diary, and 35 patients who did not perform UFM were excluded from analysis. Finally, 352 male patients were included for the analysis. The study was approved by the Institutional Review Board of Boramae Medical Center.

### Patient evaluation

The medical history of every patient was recorded, including the presence of diabetes mellitus and hypertension and previous use of any medication affecting LUTS (S1 Table). In addition, the International Prostate Symptom Score (I-PSS) questionnaire was administered at the initial visit. Serum prostate-specific antigen levels, urine characteristics, and prostate volume were measured using transrectal ultrasonography. In addition, UFM with PVR was performed at the subsequent visit, which was scheduled at 1–2 weeks after the initial visit. Patients were generally asked to hold their urination for 3–4 h before UFM to achieve sufficient VV. If this requirement was not met before presenting to the clinic, the patients were asked to drink a few cups of water and hold their urination at least 1 h before UFM. In addition, immediately before UFM, patients were asked if they feel the urge to void, and UFM was generally performed only if urinary urgency was present. Data from UFM was primarily recorded by the physician assistant, who was blinded to clinical information, with the help of the clinicians. The uroflowmeter used for the current study was Portaflow advanced v2.0.6.B (Bratain, Mediwatch).The FVC was routinely completed for 72 h. [8]

### Outcomes

Patients were divided into four groups based on the VV on initial UFM (1st quartile vs. 2nd quartile vs. 3rd quartile vs. 4th quartile). Each patient’s bladder voiding efficiency (BVE) was calculated using the following formula: BVE = VV/(VV + PVR) × 100.[9] On the Basis of on the I-PSS questionnaire, the voiding and storage subscores were calculated. The total number of voids, number of nocturnal voids, maximum voided volume (MVV), 24-h urine output (UO), and nocturnal UO were derived from the FVC. On the basis of these variables, the nocturnal polyuria index and nocturnal bladder capacity index were calculated.[10] In addition, the mean daytime UO per void was calculated as follows: mean daytime UO per void = (24-h UO—nocturnal UO)/number of daytime voids. Data were anonymized prior to access and analysis.

### Statistical analysis

Patients’ characteristics were presented and compared among the groups as means ± standard deviations for continuous variables and frequency tables for categorical variables. In addition, the groups were compared in terms of 1) I-PSS and 2) FVC-derived information. Furthermore, to estimate the probability of obtaining a result of VV ≥ 150 mL on daytime examinations, we evaluated the proportion of patients in each group with a mean daytime UO per void ≥ 150 mL. Univariate and multivariate linear regression analyses were performed to determine which demographic and FVC-derived variables affected the VV on initial UFM. Variables with a *p-*value < 0.2 in the univariate analysis were included in the multivariate analysis, in which the backward elimination method was used. All statistical comparisons were performed using IBM SPSS version 21 (IBM SPSS, Armonk, NY, USA). A *P*-value < 0.05 was considered statistically significant.

## Results

A total number of 352 patients were included in this study and mean age was 63.6 years. The mean VV was 183.0 mL, mean I-PSS was 17.4, mean storage subscore was 7.1 and mean voiding subscore was 10.3. VV decreased as the patients’ age increased (67.5 vs. 66.3 vs. 63.8 vs. 58.9 years; p < 0.001 (Table 1)). Prostate volume also differed according to VV (34.8 vs. 37.4 vs. 38.5 vs. 28.4 cm^3^; p = 0.002) although no specific trends could be identified.

**Table 1:** Patient characteristics according to voided volume on initial uroflowmetry.

	< 68.8 mL	≥68.8 and <150 mL	≥150 and <250 mL	≥ 250 mL	*p*
Number of patients, n	88	89	87	88	
Age, years, mean ± SD	67.5 ± 10.5	66.3 ± 10.6	63.8 ± 10.2	58.9 ± 10.7	<0.001
BMI, kg/m^2^, mean ± SD	24.0 ± 3.1	24.2 ± 2.8	24.2 ± 2.7	24.2 ± 3.0	0.933
Diabetes mellitus, n (%)	18 (20.0)	19 (21.1)	17 (18.5)	11 (12.2)	0.409
Hypertension, n (%)	36 (40.0)	36 (40.0)	30 (32.6)	25 (27.8)	0.234
Previous medication affecting LUTS, n (%)	32 (35.6)	24 (26.7)	18 (19.6)	20 (22.2)	0.074
Prostate specific antigen, ng/mL, mean ± SD	3.0 ± 3.5	2.6 ± 3.8	3.5 ± 6.2	2.1 ± 5.8	0.330
Prostate volume, cc, mean ± SD	34.8 ± 14.5	37.4 ± 20.6	38.5 ± 20.6	28.4 ± 10.9	0.002
Uroflowmetry, mean ± SD					
Peak flow rate, mL/sec	4.8 ± 2.2	9.1 ± 3.8	13.1 ± 4.3	19.9 ± 8.7	<0.001
Voided volume, mL	39.8 ± 18.9	109.8 ± 23.1	192.8 ± 25.8	364.4 ± 109.4	<0.001
Post voided residual urine, mL	26.7 ± 57.0	19.5 ± 33.9	22.1 ± 40.1	16.8 ± 20.6	0.405
Bladder voiding efficiency, %	73.3 ± 27.3	87.7 ± 15.1	91.5 ± 10.8	95.7 ± 5.0	<0.001
I-PSS, mean ± SD					
Sum of score	18.0 ± 8.4	18.6 ± 7.7	16.7 ± 8.1	16.7 ± 7.4	0.319
Voiding sub-score	10.2 ± 5.8	10.8 ± 5.5	9.9 ± 6.1	10.2± 5.5	0.778
Storage sub-score	7.8 ± 3.8	7.8 ± 3.7	6.8 ± 3.5	6.2 ± 3.4	0.010
Quality of life index	4.0 ± 1.3	4.1 ± 1.2	4.0 ± 1.0	3.7 ± 1.1	0.131

All other patient characteristics were equivalent regardless of VV on initial UFM. The urinalysis results, including microscopic hematuria, pyuria, and nitrite positivity, were also similar among the groups. Among the UFM-derived parameters, Qmax (4.8 vs. 9.1 vs. 13.1 vs. 19.9 mL/s; p < 0.001) and BVE (73.3 vs. 87.7 vs. 91.5 vs. 95.7%; p < 0.001) significantly decreased as VV decreased, although the PVRs were similar. The total I-PSS, voiding subscore, and quality-of-life index were similar among the groups. Conversely, the storage subscore was significantly higher in patients with lower VV on initial UFM (7.8 vs. 7.8 vs. 6.8 vs. 6.2; p = 0.010).

**Fig 1:**
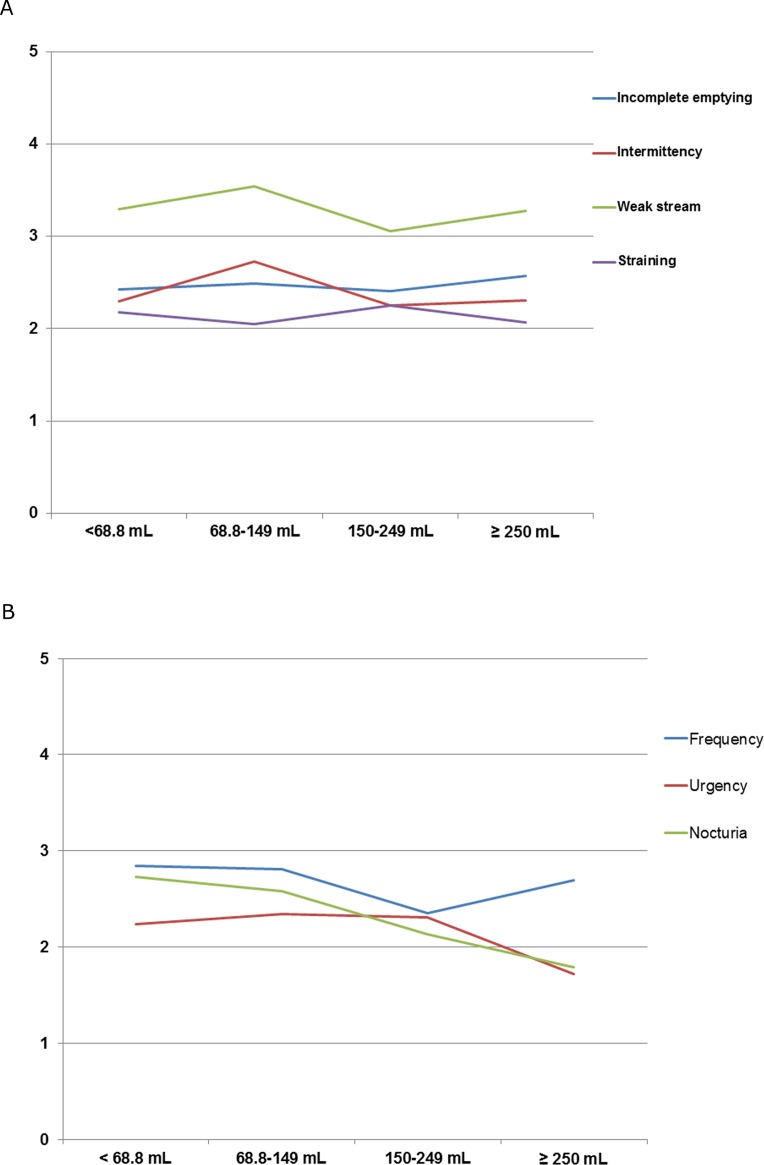
Each score in I-PSS questionnaire according to voided volume on initial uroflowmetry. (a) Question related to voiding sub-score (Incomplete emptying: *p* = 0.930, Intermittency: *p* = 0.287, Weak stream: *p* = 0.363, Straining: *p* = 0.887). (b) Question related to storage sub-score (Frequency: *p* = 0.174, Urgency: *p* = 0.079, Nocturia *p*<0.001).

The factor used to calculate the voiding subscore, including incomplete emptying (2.4 vs. 2.5 vs. 2.4 vs. 2.6; p = 0.930), intermittency (2.3 vs. 2.7 vs. 2.3 vs. 2.3; p = 0.287), weak stream (3.3 vs. 3.5 vs. 3.1 vs. 3.3; p = 0.363), and straining (2.2 vs. 2.1 vs. 2.3 vs. 2.1; p = 0.887), were similar according to VV on initial UFM (Fig 1). Among the parameters used to calculate the storage subscore, nocturia (2.7 vs. 2.6 vs. 2.1 vs 1.8; p < 0.001) significantly increased as the VV on the initial UFM decreased. Conversely, neither frequency (2.8 vs. 2.8 vs. 2.4 vs. 2.7; p = 0.174) nor urgency (2.2 vs. 2.3 vs. 2.3 vs. 1.7; p = 0.079) was associated with VV on initial UFM.

Among the FVC-derived information, the total number of voids (9.5 vs. 9.7 vs. 8.3 vs. 8.0; p < 0.001) and number of nocturnal voids (1.7 vs. 1.7 vs. 1.6 vs. 1.1; p = 0.007) were significantly higher in patients with decreased VV on initial UFM (Table 2). The MVV (314.5 vs. 331.6 vs. 377.8 vs. 458.7 mL; p < 0.001) and 24-h UO (1460.6 vs. 1657.5 vs. 1719.6 vs. 1793.5 mL; p = 0.003) continuously decreased as VV on initial UFM decreased. Nocturnal UO, Nocturnal polyuria index, and nocturnal bladder capacity index were similar regardless of VV on initial UFM. The mean daytime UO per void (127.8 vs. 142.2 vs. 175.1 vs. 185.6 mL; p < 0.001) significantly decreased as VV on initial UFM decreased. The proportion of patients with a mean daytime UO per void ≥ 150 mL were 30.1%, 43.0%, 64.7%, and 66.7% in each group, respectively.

**Table 2:** Information derived from frequency volume chart according to voided volume on initial uroflowmetry.

	< 68.8 mL	≥68.8 and <150 mL	≥150 and <250 mL	≥ 250 mL	*p*
Number of total voids, n, mean ± SD	9.5 ± 3.4	9.7 ± 3.4	8.3 ± 2.3	8.0 ± 2.6	0.001
Number of nocturnal voids, n mean ± SD	1.7 ± 1.1	1.7 ± 1.2	1.6 ± 1.4	1.1 ± 1.1	0.007
Maximum voided volume, mL, mean ± SD	315 ± 122	332 ± 111	378 ± 149	459 ± 158	<0.001
24-hour urine output, mL, mean ± SD	1461 ± 693	1658 ± 542	1720 ± 605	1794 ± 588	0.003
Nocturnal urine output, mL, mean ± SD	493 ± 265	538 ± 255	558 ± 288	567 ± 313	0.303
Nocturnal polyuria index (NPi), mean ± SD	34.5 ± 13.7	32.2 ± 12.9	32.4 ± 12.6	31.1 ± 12.8	0.362
Predicted nocturnal voiding (PNV), mean ± SD	0.6 ± 0.7	0.7 ± 0.9	0.5 ± 0.8	0.3 ± 0.7	0.001
Nocturnal bladder capacity index (NBCi), mean ± SD	1.0 ± 0.7	1.0 ± 0.6	1.0 ± 1.1	0.8 ± 0.9	0.446

On univariate analysis, patient age, prostate volume, 24-h UO, and MVV were significantly associated with VV on initial UFM (Table 3). On multivariate analysis, older age (B, -3.495, p < 0.001) and decreased MVV (B, 0.350, p < 0.001) emerged as significant variables associated with decreased VV on initial UFM in men with LUTS.

**Table 3:** Variables affecting voided volume on initial uroflowmetry.

	Univariate				Multivariate			
		95% CI				95% CI		
	B	Lower	Upper	p	B	Lower	Upper	p
Age	-4.032	-5.250	-2.814	<0.001	-3.495	-4.889	-2.100	<0.001
BMI	1.522	-3.362	6.405	0.540				
Diabetes	-19.87	-56.19	16.45	0.283				
Hypertension	-29.61	-58.94	-0.279	0.048				
Prostate volume	-1.187	-2.067	-0.307	0.008				
24-hour urine output	0.034	0.012	0.057	0.003				
Nocturnal urine output	0.020	-0.030	0.071	0.434				
Maximum voided volume	0.366	0.277	0.454	<0.001	0.350	0.253	0.446	<0.001

## Discussion

UFM is one of the most frequently performed examinations to evaluate LUTS in men, and a VV < 150 mL in UFM is regarded as an incomplete test result in assessing UFM-derived parameters. [11] However, the present study suggested that a VV of ≥ 150 mL on initial UFM cannot be achieved by about 50% of men with storage symptoms, although these symptoms are reported to be common in adult men. [12] Moreover, about one-third of patients demonstrated a VV < 100 mL on initial UFM. In other words, initial UFM could provide less information owing to insufficient VV in a considerable number of men with storage symptoms. However, the present results indicate that clinicians could use the VV on initial UFM to predict the severity of storage symptoms, although the volume is < 150 mL. To our knowledge, the present study is the first to suggest an objective relationship between VV on UFM and storage symptom severity.

In the present study, subjective storage symptoms were more severe as VV on initial UFM decreased, although the total I-PSS and voiding subscores were equivalent. Moreover, patients with decreased VV on initial UFM actually had increased number of voids, although the 24-h UO was significantly decreased. In a previous study, storage symptoms increased as fluid intake increased, [13] and decreased fluid intake, which resulted in decreased UO, could be incorporated into the patients’ multifarious efforts to alleviate their symptoms. [14] That is, patients with decreased VV on initial UFM were believed to have severe storage symptoms despite their efforts to alleviate those symptoms. Thus, decreased VV on initial UFM could be considered a sign of severe storage symptoms in daily clinical practice. Moreover, on the basis of the present study, decreased VV on initial UFM may be caused by older age or decreased functional bladder capacity. With respect to older age, it was possible that a greater proportion of older patients did not understand the requirement to sufficiently delay urination before the examination, and this may have resulted in the decreased VV on initial UFM in elderly patients. Conversely, increased age has been associated with detrusor overactivity, [15] which may be another reason for the association between older age and decreased VV on initial UFM. Decreased functional bladder capacity has also been associated with LUTS severity. [16]

The mean daytime UO per void was lower in patients with decreased VV on initial UFM in the present study. In addition, the mean daytime UO per void was lower than that in adults without LUTS, as reported in a previous study. [17] In other words, we could hypothesize that the VV on initial UFM might be associated with the mean daytime UO per void because, importantly, most examinations, including UFM, tend to be performed during daytime. Therefore, researchers sensibly consider the mean daytime UO per void as a surrogate predictor of VV on repeat UFM. The current study suggests that more than half of patients with a VV < 150 mL on initial UFM have a mean daytime UO per void of < 150 mL. Moreover, as VV on initial UFM decreased, the probability that the same patient would have a VV ≥ 150 mL on repeat UFM decreased. Moreover, because the correlation coefficient between VV on UFM and storage sub-score was -0.153 (p = 0.005), which was similar to that between mean daytime UO per void and storage sub-score of -0.144 (p = 0.009), VV on UFM was believed to have at least similar value for predicting storage sub-score compared with mean daytime UO per void. However, these are only hypothesis-generating and remained to be further validated in the future study.

In this regard, decreased VV on UFM should not be ignored by clinicians, and the probability of severe storage symptoms should be carefully assessed. In other words, the presence of overactive bladder in patients with decreased VV on UFM need to be suspected by clinicians, and it is better to perform a careful clinical assessment to diagnose and treat overactive bladder. In addition, in such patients, repeating UFM after the initiation of medical treatment, such as antimuscarinic agent and/or β3-agonist administration, which reportedly increases the VV may be useful [18]. In addition, performing another examination, such as FVC assessment, may be more appropriate than conducting repeat UFM in these patients. Although FVC assessment is currently recommended for patients with storage symptoms, completing a 72-h FVC takes time and effort. Because UFM is recommended as a routine evaluation for patients with LUTS, our results could be useful for appropriately selecting patients with storage symptoms, who are eligible for FVC assessment. In other words, VV on UFM could also be used as a tool to screen patient for the need for FVC assessment, in addition to its use for distinguishing outflow obstruction.

Although a decreased VV on initial UFM may be a sign of severe storage symptoms, as mentioned above, the current study suggests that the parameters derived from UFM with decreased VV are less informative for clinicians. Specifically, Qmax and BVE seemed to significantly decrease as VV on initial UFM decreased, as was found in a previous study. [3] Conversely, PVR was similar among the groups, and therefore the probability of chronic retention was not increased even when VV on initial UFM is decreased. Nevertheless, because the utility of PVR in predicting urinary retention is still controversial, [19, 20] we cannot conclude that decreased VV on initial UFM predicts a similar risk of acute urinary retention when antimuscarinic agents are prescribed. It follows that treatment must be initiated after consideration of patient characteristics and risk of urinary retention.

This study had several limitations in addition to its retrospective design. First, several variables that can affect VV on initial UFM were not assessed in this study. In a previous study, psychological status was reported to affect tolerance to bladder filling. [21] Moreover, because the intervoid interval before UFM was not recorded, it cannot be adjusted and the impacts of intervoid interval remain to be analyzed in a future study. We could not quantify the degree to which the clinicians explained the FVC procedures or whether the patients understood the requirement for holding their urine before UFM. As mentioned above, the degree of understanding and explanation might be related to the impact of age on decreased VV and this needs to be evaluated in a future study. Another limitation is existence of a selection bias. In the present study, only patients who completed the FVC were included in the analysis. Thereby, patients with severe storage symptoms may have been selected, and the results of the current study may be applicable only to men with storage symptoms. The last limitation of the current study is that the role of repeat UFM in patients with decreased VV on initial UFM has not been checked, and this should be investigated in a future study. Nevertheless, to our knowledge, this was the first study to evaluate the clinical role of VV on initial UFM in assessing the severity of LUTS, especially storage symptoms. Moreover, our results might be clinically useful in managing patients in daily practice, because we selected patients at their first visit to our urology department and excluded patients whose storage symptoms had other causes.

## Conclusions

A decreased VV on initial UFM may be a sign of severe storage symptoms in men with LUTS, a condition that is related to older age and decreased functional bladder capacity. In these patients, it is better to perform a careful clinical assessment to diagnose and treat overactive bladder. In addition, repeat UFM should be performed after treatment is initiated to ensure that a VV of ≥ 150 mL is achieved on the repeat examination.

## Supporting information

S1 Table Medications which affect lower urinary tract symptoms. (DOCX)
