# Supplementary material for: Voided volume < 150 mL on initial uroflowmetry in men with storage symptoms: Is it an unreliable test result or a sign of severe storage symptoms?
Source: PLoS One. 2019 Jan 7;14(1):e0207208. doi: 10.1371/journal.pone.0207208 (PMC6322755; doi:10.1371/journal.pone.0207208)
Supplement: S1 Table — (DOCX) [file pone.0207208.s001.docx]

S1 Table. Medications which affect lower urinary tract symptoms

| α-blocker | Alfuzosin  Doxazosin  Silodosin  Tamsulosin  Terazosin |
| --- | --- |
| 5α—Reductase Inhibitors | Dutasteride  Finasteride |
| anticholinergics | Darifenacin  Oxybutynin  Solifenacin  Trospium  Tolterodine |
| β3-agonist | Mirabegron |
